# The Investigation of the Production of Salt-Added Polyethylene Oxide/Chitosan Nanofibers

**DOI:** 10.3390/ma17010132

**Published:** 2023-12-27

**Authors:** Sandra Varnaitė-Žuravliova, Natalja Savest, Julija Baltušnikaitė-Guzaitienė, Aušra Abraitienė, Andres Krumme

**Affiliations:** 1 Department of Textile Technologies, Center for Physical Sciences and Technology, Demokratų Str. 53, LT-48485 Kaunas, Lithuania; julija.baltusnikaite@ftmc.lt (J.B.-G.);; 2 Department of Materials and Environmental Technology, Tallinn University of Technology, Ehitajate Tee 5, EE-19086 Tallinn, Estonia; natalja.savest@taltech.ee (N.S.); andres.krumme@taltech.ee (A.K.)

**Keywords:** electrospinning, nanofibers, salt, chitosan, polyethylene oxide

## Abstract

The influence of different concentrations of salt-added polyethylene oxide (PEO) on the spinnability of chitosan (CS)/PEO + NaCl blends that could be used as a component part of filters for water treatment or nanofiber membranes as well as for medical applications was investigated in this study. The morphological properties of manufactured nanofibers were analyzed as well. It was determined that an increase of PEO concentration resulted mostly in thin and round nanofibers formed during electrospinning, but the manufacturing process became complex, because many wet fibers reached the collector while spinning. Also, it was noticed that the salt was not dissolved completely in the polymer solutions and some crystals were seen in the SEM images of manufactured fiber mats. However, the addition of salt resulted in lower viscosity and better conductivity of solution and fiber mats as well. The opposite effect was observed as the concentration of PEO was increased. The orientation of produced nanofibers as well as their diameter were analyzed with commercially available software. It was determined that the results obtained by software and microscopically are repeatable. The difference among the results of diameter calculated with software and taken by microscope varied from 0% to approximately 12%. The FTIR analyses indicated that alterations in polymer concentrations or the addition of salt did not induce any discernible changes in the chemical composition or nature of the materials under investigation. The sodium chloride present in the solutions enhanced electrical properties and increased conductivity values more than 50 times for PEO solutions and six times for CS/PEO blend solutions, compared to conductivity values of solutions without salt. To assess the thermal characteristics of the PEO/CS blend nanofibers, measurements using a differential scanning calorimeter (DSC) to determine melting (Tm) and crystallization (Tc) temperatures, as well as specific heat capacities were conducted. These parameters were derived from the analysis of endothermic and exothermic peaks observed in the DSC data. It showed that all produced nanofibers were semicrystalline.

## 1. Introduction

Chitosan (chitosan) is currently receiving a great deal of interest not only in medical and pharmaceutical application but also as a promising candidate for use as an absorbent system for various common and emerging pollutants such as heavy metals, anionic organic dyes, and anti-inflammatory drugs [1,2]. In recent studies, considerable attention has been directed towards polymer nanoparticles as a vehicle for drug administration. Nanoparticles play a distinct role in targeted drug delivery by possessing advantageous features akin to liposomes, particularly their size characteristics. On the other hand, not only the form of chitosan but also its derivatives may improve the efficiency of drug delivery [3]. However, in contrast to liposomes, nanoparticles exhibit extended stability over time and possess superior drug entrapment capabilities. The existence of amino and hydroxyl groups within the chitosan polymer structure enables the chelation of metals, facilitating their adsorption from aqueous solutions [4,5].

Chitosan is a biosynthetic polysaccharide—the only polycation in nature whose charge density depends on the degree of acetylation and pH of the media [6,7]. Our interest has been focused on complexes of biosynthetic and synthetic polymers—chitosan and polyethylenoxide (PEO). PEO is a large molecule made from linking together ethylene oxide units through a process called polymerization. PEO comes in high-polymer form, characterized by molecular weights that typically range from 150,000 to 10 million.

The combination of CS and PEO polymers leads to the creation of colorless materials. In this blend, CS contributes to enhanced mechanical properties and reduced solubility in water, while PEO contributes to the development of products with heightened flexibility. Recent studies indicate that the resulting composite nanofibrous scaffolds exhibit stability, do not exhibit toxicity to cells, and are environmentally sustainable [8,9,10].

The distinctive amphiphilic nature and unique mechanical attributes of PEO allow successful electrospinning of CS blends with PEO into nanofibers.

Numerous researchers are currently exploring the production methods of nanofibers derived from chitosan (CS) and its combinations, particularly with high molecular weight polyethylene oxide (PEO) [4,6,8,9,10,11,12,13]. However, despite these efforts, accurately anticipating the specific traits of these polymers and their behavior during the nanofiber manufacturing process remains a challenging task.

Additives are employed to modify various properties of the solution, including viscosity, electrical conductivity, dielectric strength, and viscoelastic characteristics [14,15]. It is well known that the behavior of polymer macromolecules changes after the addition of salt. Sodium chloride (NaCl) is frequently added to aqueous solutions as an additive. Its primary function is to disrupt hydrogen bonding within the solution, thereby aiding in the enhancement of dissolution processes [14,15]. At lower concentrations of salt, the chitosan chains experience increased stretching and dispersion due to electrostatic repulsion. This phenomenon facilitates the flexible PEO chains to readily form molecular complexes by engaging in hydrogen bonding with chitosan [16]. The introduction of a low molecular weight salt into the spinning solution induces changes in solution conductivity, as ions function as charge carriers within an electric field. This alteration influences the motion of the spinning jet, consequently impacting the morphology of the electrospun materials and the resulting fiber structure [14,15,17,18]. With an increase in concentration of the added salt, the effect of charges residing on chains diminishes and the chitosan chains become coiled, resulting in a decrease in contribution of the bonds between hydrogen atoms of the quaternary ammonium group of chitosan and the ether oxygen of PEO, leading to the creation of intramolecular hydrogen bonds. Consequently, the strength of interaction between PEO and chitosan chains diminishes [6,19]. A threshold concentration of NaCl exists beyond which a salting-out effect becomes apparent. This phenomenon might arise due to the disruption of interactions between the solute and solvent molecules at elevated NaCl concentrations. However, in the case of electrolytes, the salt also affects conformation of the macromolecules and therefore their interactions with the environment [6,7,16,20]. Chitosan is also rigid as a result of its intramolecular hydrogen bonds [16]. The solution properties (mostly viscosity) changes during the shrinkage of the macromolecule as a consequence of interaction of dissociated NaCl and chitosan polycation [6,20].

Angammana et al. in [21] observed a close correlation between solution conductivity and average jet current, noting an initial rise in jet current with increasing solution conductivity, followed by a subsequent decrease at higher conductivity levels. They established a power relationship where increased solution conductivity correlated with decreased fiber diameter. These outcomes stem from the distribution of electric charge on the solution droplet’s surface and the applied tangential field. Their study employed NaCl to enhance solution conductivity, which interferes in two ways: firstly, by augmenting the number of free ions migrating towards the droplet’s surface, thereby elevating surface charge density, and secondly, by boosting solution conductivity, consequently reducing the applied tangential electric field on the solution’s surface [21]. Typically, the introduction of small amounts of salts into electrospinning solutions is recognized for its role in reducing the formation of beads by amplifying the overall charge density on the surface of the jet [22]. In a separate study, Jiang et al. introduced varying quantities of an organic ammonium salt into a PEO–chloroform solution to manipulate its conductivity and consequently the free charge density. The findings demonstrated that as conductivity increased, the angle of the envelope cone of the jet also increased. This change made it more challenging to deposit aligned fibers [20].

Electrospinning is a versatile technique capable of generating innovative fibers within a wide diameter spectrum, spanning from 100 nanometers to 10 μm. The process involves the use of an electric field to draw a polymer solution or melt into ultrafine fibers. The basic electrospinning setup consists of a syringe or reservoir containing a polymer solution or melt, a metallic needle or spinneret connected to a high-voltage power supply, and a grounded collector plate or rotating drum. A polymer, such as polyethylene oxide (PEO), chitosan (CS), or a blend of polymers, is dissolved in a solvent to create a homogeneous solution. The polymer solution is then loaded into a syringe connected to the needle (spinneret). When a high voltage is applied to the needle, it induces electrostatic charges on the surface of the polymer droplet at the needle tip. The repulsion of charges causes the formation of a Taylor cone, a cone-shaped droplet at the needle’s end. As the electrostatic repulsion overcomes the surface tension of the polymer solution, a fine liquid jet, known as the Taylor cone jet, is ejected from the needle’s tip toward the grounded collector. The elongation of this jet occurs due to the electrostatic forces applied. During flight to the collector, the solvent evaporates from the jet, leading to the solidification of the polymer fibers. The fibers are collected on the grounded collector.

By regulating process parameters, the size, distribution, and porosity of fiber mats can be substantially altered. For biomedical applications, drugs and growth factors can be seamlessly integrated into the structure. During solvent evaporation, polymer molecules may converge by undergoing either phase separation via a spinodal reaction or through the conventional nucleation and growth of the crystalline phase [23]. The polymer structure deposited on the collector may exhibit various forms: completely amorphous, oriented, spherulitic, or textured fibrillar structures. The level of molecular alignment within the amorphous regions directly relates to the extent of extension flow. Electrospinning has the potential to reduce the polymer’s crystallinity. During electrospinning, the jets are elongated along their axis by the external electric field and further stretched due to the repulsion between charges present on adjacent segments [24].

The objective of this investigation was to meticulously examine the impact of varying concentrations of salt-added polyethylene oxide (PEO) on the morphology, chemical composition, and thermal properties of produced chitosan CS/PEO + NaCl complexes. The innovative look at assessing the morphological characteristics of manufactured nanofibers, including factors such as orientation, directionality, and diameter, holds significant importance during this research work. These parameters notably influence various permeability properties and are pivotal in their application and utility. Nanofiber mats can serve as media, allowing the penetration of substances such as air or gases, UV light, water, various liquids, and more. Understanding and evaluating these morphological features are crucial in comprehending the performance and potential applications of these nanofibers [1,2,25].

Numerous methods exist for assessing the morphological parameters of materials, yet no single method yields universally satisfactory results across all application areas. Each evaluation technique comes with its strengths and limitations, making it essential to choose or combine methods tailored to specific requirements and applications. The selection often depends on the desired parameters, accuracy, resolution, and the material’s characteristics under investigation.

## 2. Materials and Methods

The primary chemical material employed in the research was chitosan, represented by the chemical formula (C_6_H_11_NO_4_)n, with a molecular weight falling within the range of 100,000 to 300,000 g/mol. This chitosan was procured from ACROS Organics^TM^ (Geel, Belgium).

The formulation process involved blending 99.8% acetic acid (CH_3_CO_2_H) with a molecular weight of 60.05 and a density of 1.05 kg/L with deionized water to achieve concentrations of 90% and 50%, respectively. Subsequently, a 3 wt% solution of chitosan was dissolved separately in both the 90% and 50% acetic acid solutions.

The Polyethylene oxide (PEO) utilized in the research, having a molecular weight of 200,000 g/mol, was procured from Ontario Inc. (Toronto, ON, Canada). To prepare solutions, PEO was dissolved in deionized water, resulting in concentrations of 10 wt%, 15 wt%, and 20 wt%. Additionally, sodium chloride (NaCl) was incorporated into the PEO solutions at a concentration of 0.24 mol/L. This addition aimed to elevate the electrical conductivity of the spinning solution and improve the technological aspects of the electrospinning process.

All prepared solutions underwent mechanical stirring for a duration of 24 h, followed by a degassing process lasting 4 h at room temperature. This method ensured proper mixing and removal of entrapped air or gases from the solutions before further processing or utilization.

To create blend solutions for electrospinning, the chitosan solution was combined with PEO/NaCl solutions and allowed to mix for 24 h. The mass ratio chosen for blending chitosan with PEO/NaCl was 50:50. It is important to note that all samples of chitosan and PEO/NaCl were used in their original state without undergoing additional purification processes.

Coding of prepared solutions and produced fiber mats are presented in Table 1.

For comparison purposes of FTIR analysis, fiber mats were produced from a solution of 20 wt% PEO in water and a blend solution of 3 wt% chitosan dissolved in 90% acetic acid and mixed with 20 wt% PEO in water (without NaCl), with a mass ratio of 50:50.

### 2.1. Solution Testing Equipment

The viscosity of all the prepared spinning solutions was assessed using a rotating viscometer (Brookfield, DV-II, Middleboro, MA, USA) under identical shearing rates. Simultaneously, the electrical conductivity was determined with a conductivity meter (Mettler Toledo) under standard conditions of room temperature (22 °C) and a relative humidity of 35%. The reported result represents the mean value obtained from five measurements, and the standard deviation was less than 2, indicating a low variance among the measurements.

### 2.2. Electrospinning

**Figure 1:**
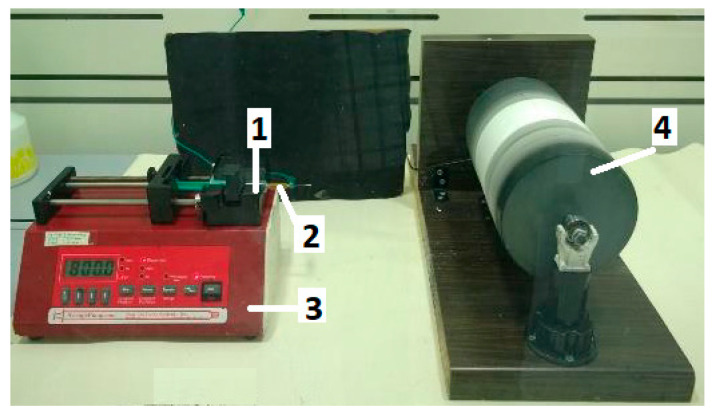
The electrospinning set-up: (1) polymer solution; (2) syringe with a metal capillary; (3) syringe pump; (4) collector drum.

The electrospinning apparatus utilized for nanofiber production is illustrated in Figure 1. A syringe (1) containing 3 mL of the polymer solution was equipped with a metal capillary (2) of 0.6 mm diameter. This capillary was connected to a high-power supply capable of generating positive DC voltage, reaching up to 40 kV. Control over the solution’s flow rate was managed by a syringe pump (3). A grounded aluminum foil was situated on the collector drum (4). The electrospun fibers were typically obtained under specific parameters: a voltage of 20 kV, a distance of 15 cm between the capillary and collector, a drum rotation speed of 320 rpm, and an adjusted flow rate of 0.5 µL/h during electrospinning. The electrospinning process occurred within a closed airflow chamber at room temperature (22 °C) and a relative humidity of 35%.

The code of the produced fiber mats corresponds to the code of the spinning solution, as shown in Table 1.

### 2.3. Characterization

The morphology of the electrospun fiber mats was examined using a scanning electron microscope (SEM), specifically the Hitachi TM-1000 model (Hitachi Ltd., Tokyo, Japan). Fiber diameter measurements were conducted utilizing commercial software on scanning electron micrographs captured at an original magnification of 3000×.

The diameter of produced nanofibers was also calculated using the computer software ImageJ (National Institute of Mental Health, Bethesda, MA, USA, https://imagej.net/ij/download.html, accessed on 1 September 2023). SEM images were captured specifically for segmentation purposes using the aforementioned software. The orientation of fibers was calculated using OrientationJ and DirectionalityJ plugins of ImageJ software. The Fourier components analysis method was used for directionality determination. The segmented SEM images, computed with ImageJ software, are presented in Figure S1 (Segmented SEM images for the determination of fiber diameter distribution with ImageJ). The segmented images, used for the analysis of nanofiber diameter, orientation, and directionality in the sample PEO10 NaCl are presented in Supplementary Materials.

The FTIR spectra were acquired employing a Perkin-Elmer PE Spectrum-GX FT-IR ATR, FT-NIR Infrared spectrometer (Perkin-Elmer, Shelton, CT, USA). Spectral data were recorded within the range of 400–4000 cm^−1^ with a resolution of 4 cm^−1^, using 32 scans to enhance the accuracy of the measurements.

To assess the thermal characteristics of the PEO + NaCl/CS blend nanofibers, various measurements were conducted using a Differential Scanning Calorimetry (DSC) instrument, specifically the Q10 model from TA Instruments, New Castel, DE, USA. This DSC setup was equipped with a refrigerated cooling system and operated under a nitrogen atmosphere at a flow rate of 50 mL/min. The DSC analyses involved subjecting specimens to heating and cooling cycles within a temperature range spanning from −50 °C to 180 °C at a heating rate of 10 °C/min^−1^. Upon reaching 180 °C, the samples were maintained for 3 min before being rapidly cooled to −50 °C. Subsequently, the samples were reheated to 180 °C.

Precisely measured samples (±0.1 mg) were positioned in an aluminum sample holder with a central pinhole and covered. Indium metal (99.99%) was utilized for the calibration of the DSC instrument with respect to both temperature and enthalpy measurements.

The calibration of the Differential Scanning Calorimetry (DSC) instrument with indium prior to the experiments was performed in order to ensure accurate temperature and enthalpy measurements during the analysis of the samples. This calibration process using indium, a known material with well-defined melting characteristics, allowed for the precise adjustment and validation of the instrument’s temperature and enthalpy readings, enhancing the reliability and accuracy of the subsequent experimental results obtained from the DSC analysis of the samples

The crystallinity degree in PEO polymer was obtained using an equation [26]:

X_c_ = ΔH_f_/ΔH_f0_ (1)

where:

X_c_—is the weight fraction extent of crystallinity, %
ΔH_f_—is the enthalpy of fusion at the melting point,
ΔH_f0_—is the enthalpy of fusion of totally crystalline polymer at the equilibrium melting point (a reference value).

A reference value of fusion enthalpy (ΔH_f0_) used for calculations of crystallinity was taken as 213.7 J/g [27].

## 3. Results

The main properties, such as electrical conductivity and viscosity, of spinning solutions, were measured (see Table 2) as soon as solutions were prepared, as all chitosan solutions change their rheological properties in time. The data presented in Table 2 indicate a noticeable pattern: an increase in the concentration of PEO in the solution led to heightened viscosity and a simultaneous decrease in electrical conductivity values. Additionally, blending chitosan with PEO resulted in a further increase in solution viscosity.

Indeed, the observed data also indicated that the concentration of acetic acid present in the samples had an influence on the conductivity values of the prepared solutions. As the concentration of acetic acid varied in the samples, it appeared to affect the electrical conductivity of the solutions. Conductivities of solutions with 90% AA are almost twice smaller than those made from 50% AA. It was expected, because other scientists received the same results [17,28,29,30,31]—increased PEO concentration resulted in enhanced solution viscosity (see Table 2). Certainly, the decrease in conductivity can be attributed to the inherent properties of PEO, considering it is a non-ionogenic polymer. PEO typically lacks ionizable groups along its molecular structure, which results in a lower ability to facilitate the movement of ions, consequently leading to reduced electrical conductivity in solution [11]. It is known from previous research [12] and literature reviews [8,9] that solutions of chitosan (CS) tend to exhibit higher conductivity compared to PEO due to the polyelectrolytic nature of chitosan. Chitosan contains positively charged amino groups along its polymer chains, imparting a polycationic characteristic. These positive charges within the chitosan structure facilitate enhanced ion mobility, leading to increased conductivity in solutions containing chitosan compared to non-ionogenic polymers like PEO. Indeed, the introduction of PEO into the chitosan (CS) solution led to a decrease in electrical conductivity. This reduction is attributed to several factors. Firstly, the formation of hydrogen bonds between the ether groups of PEO and the amino groups of CS resulted in a decrease in protonation levels. Additionally, the substitution of a positively charged molecule (CS) by a neutral one (PEO) contributed to this decrease in electrical conductivity. These alterations facilitated the creation of a stable needle jet during the electrospinning process, preventing splaying of the jet in the stretching region [8,9,11].

The sodium chloride present in the solutions enhanced electrical properties and increased conductivity values more than 50 times for PEO solutions and six times for CS/PEO blend solutions, compared to the conductivity values of solutions without salt—see Table 2 and [12]. It is noteworthy that NaCl influenced the commencement of the electrospinning process [8].

**Figure 2:**
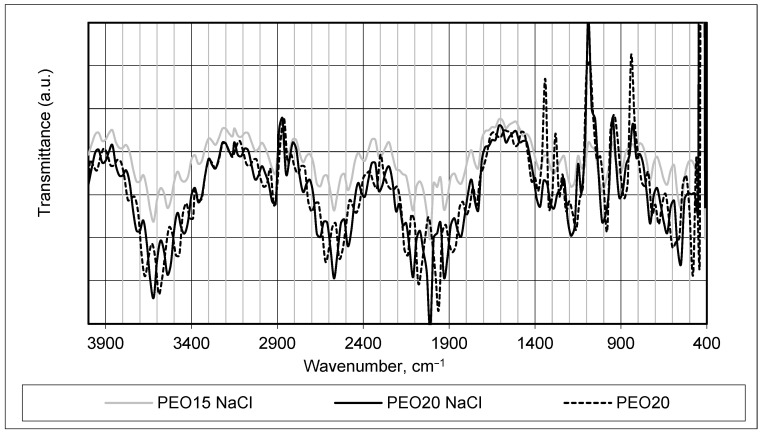
FTIR spectrum of PEO fiber mats.

**Figure 3:**
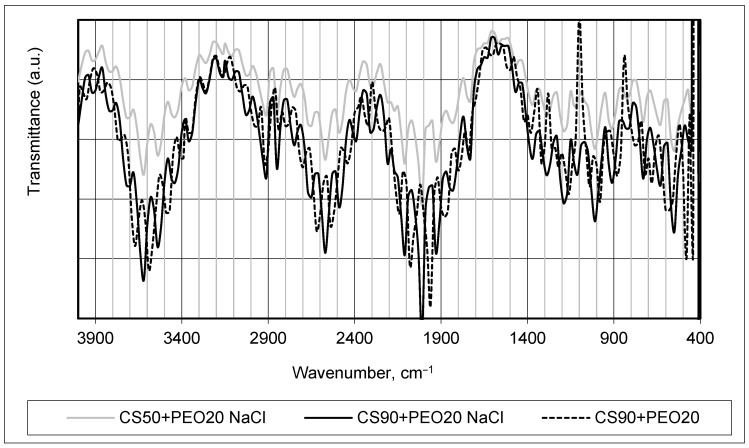
FTIR spectra of chitosan/PEO blended fiber mats.

FTIR analysis was used to monitor changes in the chemical structure of manufactured fiber mats (see Figure 2 and Figure 3). It was chosen to compare the infrared absorption spectra of produced samples PEO15 NaCl, PEO20 NaCl, and PEO15 (see Figure 2) and CS90 + PEO20 NaCl, CS50 + PEO20 NaCl, and CS90 + PEO20 (see Figure 3), and to assess side effects or extraneous compounds formed via spinning. 

The FTIR spectrum of the PEO + NaCl and pure PEO fiber mats, which is shown in Figure 2, aids in identifying alterations in the molecular structure, confirming the presence or absence of specific functional groups, and discerning any chemical modifications or interactions occurring due to the inclusion of NaCl in the PEO material. It is clearly seen that the PEO concentration resulted in changes of FTIR spectrum intensity—higher concentrations provided more obvious and sharper peaks.

A dominant band at around 3600 cm^−1^ appeared in a spectrum of a pure PEO20 sample, which was slightly shifted to the left in the spectrum of PEO samples with salt. This band was due to the hydration of PEO, and proves the high hydrophilicity of the PEO [32,33]. The PEO also showed a large band of CH2 stretching at around 2900–2600 cm^−1^ in all tested samples. Clear vibrational modes appeared in PEO in the region between 1200 and 1400 cm^−1^ which corresponded to asymmetric CH2 bending and symmetric CH2 wagging [32,33]. 

The presence of NaCl in the FTIR spectrum of samples PEO15NaCl and PEO20NaCl presented in Figure 2 was not very clearly evident. The characteristic bands of NaCl salt at around 1310 and 1110 cm^−1^ were hardly seen, and the band at 1640 cm^−1^ was not present at all [33,34]. The prominent evidence in the spectra appeared as a band around 1000 cm^−1^, primarily attributed to the symmetric and asymmetric stretching modes of vibration of C-O-C bonds present in PEO. This band underwent changes as a consequence of the addition of salt to PEO. Additionally, subtle alterations were observed in the ether oxygen band around 1850 cm^−1^ in the spectrum of PEO samples, suggesting interactions between the salt and the ether oxygen functionalities within the PEO structure. These changes in the spectral features indicate modifications in the molecular environment or interactions resulting from the presence of salt in the PEO material [34,35].

Indeed, in practice, chitosan (CS) and polyethylene oxide (PEO) form a blended mixture comprising two structurally distinct polymers. When combined, these polymers interact without establishing any covalent bond formation between their molecular structures. The interaction between CS and PEO is primarily governed by non-covalent forces such as hydrogen bonding, electrostatic interactions, and van der Waals forces, enabling them to blend and intermingle without undergoing chemical bonding [11]. The interaction between chitosan (CS) and polyethylene oxide (PEO) can involve the formation of two distinct types of hydrogen bonds: the first type of hydrogen bond can occur between the ether oxygen atoms present in PEO and the hydrogen atoms associated with the quaternary ammonium group of chitosan; the second type of hydrogen bond involves interactions between the hydrogen atoms within the hydroxyl groups of chitosan and the ether oxygen atoms in PEO. These hydrogen bond formations between the functional groups of CS and PEO contribute to their intermolecular interactions and blending without forming covalent bonds, influencing the properties and behavior of the polymer blend [6,36]. As seen in Figure 3, the major characteristic peak of chitosan associated with the amino group at around 1560 cm^−1^ (NH bond), and at approximately 3600 cm^−1^ (N-H stretching) broadband was obtainable for stretching NH2 group vibrations. The band observed around 1380 cm^−1^ in the spectrum corresponded to the stretching vibration involving OH/CH functional groups. This band indicated the presence of the OH hydroxyl group, as well as the C-O-C group, typically found around 1000 cm^−1^ in the infrared spectrum. The signals in this region provided information about the vibrational modes associated with these specific functional groups within the material being analyzed. The dominant PEO bands in the spectrum of CS/PEO samples are also seen in Figure 3.

The comparison of the FTIR spectra shown in Figure 2 and Figure 3 reveals that all the characteristic peaks corresponding to each polymer remained present, and no discernible alterations in the width or shape of these peaks were observed. This observation indicates compatibility between the polymers, suggesting that the process of forming nanofibers did not significantly impact the molecular nature of the native polymers. Furthermore, the addition of salt did not induce changes in the chemical nature of the materials employed for the investigations, as evidenced by the consistent FTIR spectra observed. The same conclusions were made in [37,38].

It is clearly seen that the acetic acid concentration resulted in changes of FTIR spectrum intensity—higher concentrations provided more obvious and sharper peaks.

**Figure 4:**
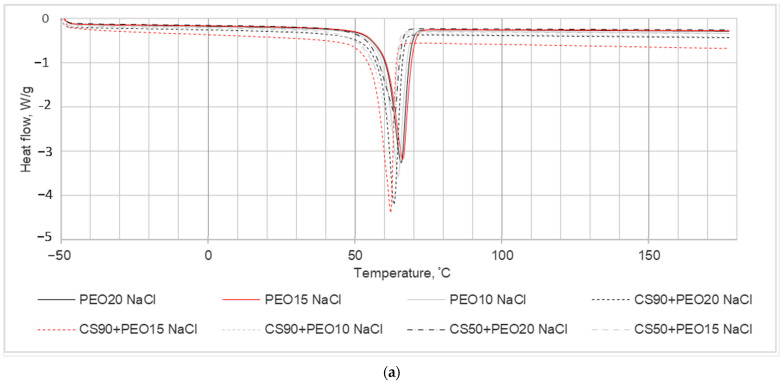

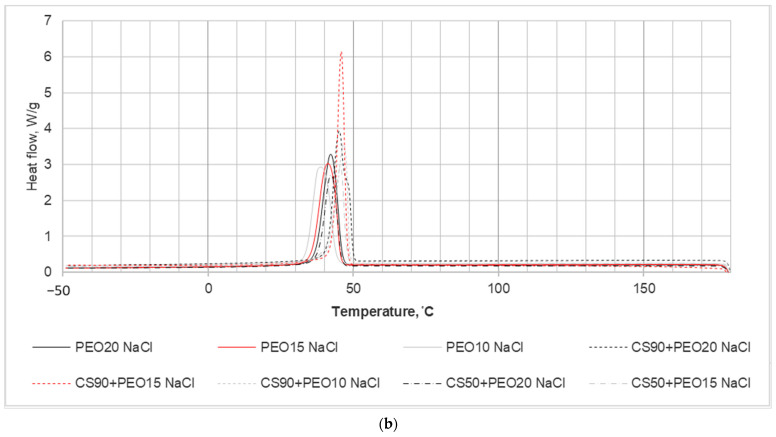
DSC crystallization thermograms of the samples tested: (**a**) second run heating cycle; (**b**) cooling cycle.

In the investigation aimed at studying the impact of chitosan (CS) presence on the crystallization kinetics of PEO nanofibrous mats, a DSC analysis was conducted, and the results are illustrated in Figure 4. The primary focus of the experiments was on the thermal properties of PEO within the blended electrospun fiber mats. The DSC analysis revealed a reduction in the enthalpy of fusion observed in the nanofiber blends compared to the pure fiber mats. This decrease in enthalpy suggests alterations in the crystallinity of the system, indicating modifications induced by the electrospinning process.

The DSC results and calculated parameters are depicted in Figure 4 and summarized in Table 3. These values were obtained from the second run of DSC scans, and the determination of melting enthalpies utilized consistent integration limits.

In the context of melting temperature (Tm), it represents the peak temperature associated with the primary endothermic peak. For pure PEO nanofibers, Tm varied between 64.7 to 66.7 °C, indicating the presence of endothermic events across all blends. Notably, as depicted in the DSC thermograms (Figure 4), an increase in PEO content within the nanofiber blends led to a shift of the PEO peaks towards higher temperatures.

Another significant observation from Figure 4 is that the melting temperature of the pure samples was higher compared to that of the blended samples. This disparity is a consequence of the presence of salts within the nanofibers, as indicated in Table 3. As additional information the crystallinity values of different concentrations of PEO solution with water are presented in Table 3.

From the thermal analysis conducted, the crystallinity percentages (Xc) of the nanofiber samples were calculated using equation No. 1. The obtained ΔHf (enthalpy of fusion) and Xc (crystallinity) values for the nanofibers are outlined in Table 3. It is evident from the table that all the nanofibers exhibited a semicrystalline nature, and the crystallinity of the nanofibers diminished with the addition of salts into the spinning solution. This decrease in crystallinity is observed to correspond with the inclusion of salts, indicating an influence of salt addition on the crystalline structure of the resulting nanofibers.

The results of thermal properties of CS/PEO and PEO nanofibers without NaCl added can be found in [12].

All attempts to produce pure chitosan nanofibers via electrospinning were unsuccessful. There can be found some literature [6,13,16,38] in which researchers state that it is impossible to spin pure chitosan fibers (in different weight ratios) using conventional coaxial electrospinning, but there are some authors who state otherwise [37,39]. In practice, the spinning process of pure chitosan is difficult due to the quite high viscosity and surface tension created in solutions with a sufficient amount of polymer. 

In order to produce chitosan nanofibers, the addition of PEO to the solutions is the easiest way. It encourages chitosan chains to form hydrogen bonds between the etheric oxide atoms in PEO and amino groups in chitosan and to reduce chitosan’s self-association effect. 

In the electrospinning process, it is beneficial for the molecular weight of chitosan to be relatively low. This is because chitosan, as a polymer, tends to create a robust network structure facilitated by the action of intermolecular and/or intramolecular hydrogen bonds. Lower molecular weight chitosan enables easier chain mobility, facilitating the formation of fibers and the development of the desired network structure during electrospinning. This lower molecular weight aids in achieving the desired properties and characteristics in the resulting chitosan-based nanofibers [6]. 

**Figure 5:**
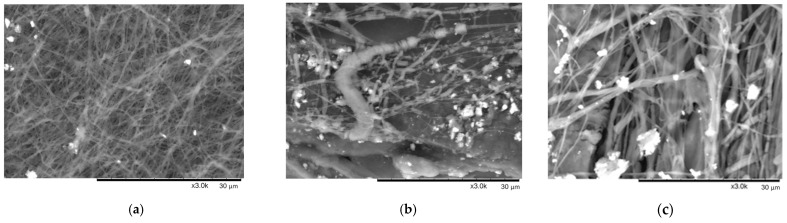

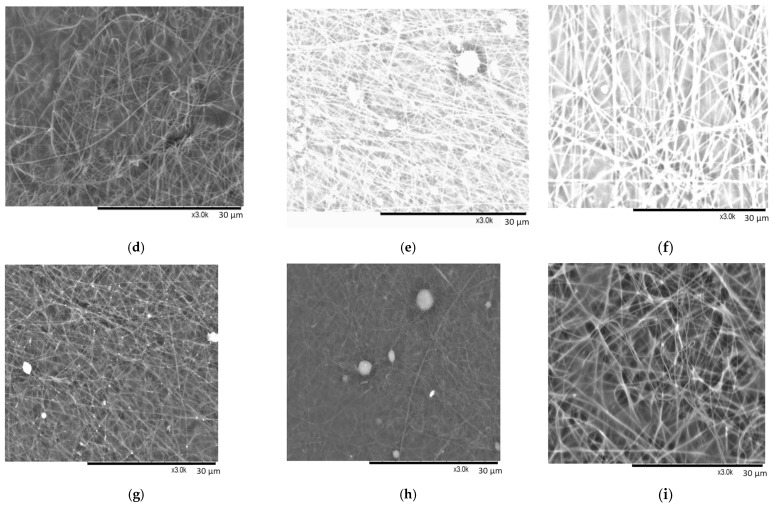
Segmented SEM images for determination of fiber diameter distribution with ImageJ: (**a**) PEO10 NaCl; (**b**) PEO15 NaCl; (**c**) PEO20 NaCl; (**d**) CS90 + PEO10 NaCl; (**e**) CS90 + PEO15 NaCl; (**f**) CS90 + PEO20 NaCl; (**g**) CS50 + PEO10 NaCl; (**h**) CS50 + PEO15 NaCl; (**i**) CS50 + PEO20 NaCl.

Indeed, the addition of NaCl to a solution typically leads to an increase in viscosity while simultaneously reducing surface tension. This addition can result in the production of finer nanofibers and a decrease in the presence of nanobeads within the resulting fiber mats. The mixing ratio of the two polymers and the inclusion of small electrolyte molecules like sodium chloride in the spinning environment significantly influence the formation of nanofibers. These effects primarily occur due to intermolecular interactions between the components present in the solution, impacting the electrospinning process and subsequent nanofiber morphology [6]. In practice, the decision to add salt to the spinning solutions gave us controversial results (see SEM images below—Figure 5).

It is seen in Figure 5a that not round but flat fibers were formed while spinning PEO10 NaCl solution. Also, we noticed that salt crystals were not fully dissolved, although solutions were mixed for 24 h prior electrospinning. Those crystals are clearly seen in Figure 5a. 

The electrospinning of solution PEO15 NaCl was quite problematic, and very thick, defected, and broken fibers were obtained (see Figure 5b). Flat and ribbon-like nanofibers were mostly formed due to the low evaporation rate of solvent and because wet fibers reached the collector while spinning. The formation of flattened fibers was described in detail by Fong and colleagues in [40]. The non-evaporated solution resulted in some kind of conglomerate mixed among fibers. Not completely dissolved salt crystals are obviously seen in Figure 5b as well.

The PEO20 NaCl nanofibers, presented in Figure 5c, look similar to fibers produced from PEO 15 NaCl: ribbon-shaped, flat, wide, and discontinuous fibers, caused by the low evaporation rate of solvent. Salt crystals are also presented in the sample. The fibers in the investigated sample look randomly oriented. 

The addition of the chitosan to the PEO/NaCl solution resulted in the electrospinning of quite round nanofibers (Figure 5d–i). The dissolved salt crystals are noticed only in Figure 5e,g. The non-evaporated solvent is obvious in all SEM images of CS + PEO NaCl samples resulting in the shadows seen in the SEM images and some nanofibers merging with each other. 

A few drops of a solvent are visible in Figure 5h, which are the result of a sprayed jet before reaching the collector. Such features of non-evaporated solvent are a key factor in such a mesh-like structure of fiber mats (see Figure 5g–i). The merging or joining of two fibers occurs through a process involving nucleation and subsequent growth. As the fibers intersect or come into contact during their formation or post-processing stages, nucleation sites facilitate the initial bonding or fusion. This process is followed by the growth of the bonding region, resulting in the merging or fusion of the individual fibers at their intersection points. Probably, if the distance between the collector and the needle would be longer, the solvent would be completely evaporated. Hohman and co-authors depicted the formation of round fibers in [41], while Gidson and colleagues characterized a mesh-like structure of nanofibers produced in [42].

Very small bead-on-string morphology in the sample CS50 + PEO15 NaCl is observed (Figure 5h). The beads often form on fibers due to the influence of the surface tension of the solution. In instances where the surface tension is considerable and substantial compared to the tangential force generated by electrical repulsion, particularly in solutions with poor conductivity, the surface tension resists the stretching force of electrical repulsion. This resistance leads to the formation of beads rather than smooth fibers, as the forces favor the bead formation over continuous stretching and stabilization of the fiber during the electrospinning process.

In conclusion, the nanofibers generated from solutions containing 50% acetic acid exhibited thinner fibers with narrower diameter distributions compared to those produced from solutions with 90% acetic acid. Higher solution viscosity led to the production of smoother and thicker fibers, attributed to increased entanglement among macromolecules within the solution. This extensive entanglement affected the fiber morphology, resulting in the formation of smoother and thicker fibers. Thinner fibers with reduced defects were observed to be produced from solutions exhibiting higher conductivity levels, as indicated by Figure 2 and SEM images displayed in Figure 5. As the electrical conductivity increased, surface charge densities also escalated, intensifying the repulsive forces between adjacent segments. This heightened repulsion effectively countered the viscous effects, amplifying elongation stress during the electrospinning process. Consequently, this interplay led to the formation of thinner fibers with fewer bead-like structures, contributing to the enhanced quality and reduced defects observed in the resulting nanofibers [43].

The morphology and relative positioning of fibrous materials can be effectively characterized by key parameters: density, average fiber diameter, and porosity. The unique and intricate structure of electrospun materials results from a multifaceted interplay of various factors. These include the composition and properties of the polymer solution utilized, the specific processing parameters employed during electrospinning, and the environmental conditions present during the fabrication process. The combined influence of these factors contributes significantly to the final morphology, properties, and structural characteristics of electrospun materials. Incorporating additives into the polymer solution enables significant modulation of electrical conductivity, viscosity (thus influencing voltage), flow rate, the formation of Taylor’s cone, and the velocity of solvent evaporation. Each of these factors profoundly impacts the characteristics of the resulting fibers, such as color, surface texture, morphology, the presence of inclusions, and the occurrence of defects [44].

The histograms of fibers’ orientation and directionality in the samples are presented in Supplementary Figure S2 (Histograms of fiber orientation in samples). The peaks in the orientation histograms provide data about the anisotropic composition of the sample structure. According to the findings presented in [37,39], a flat directionality histogram indicates a completely isotropic composition of the sample structure. In other words, it suggests that there is no preferential orientation or alignment of the components within the sample. The absence of peaks or variations in the directionality histogram implies that the structural elements are uniformly distributed and exhibit no specific directional preference or alignment. Following this, it can be stated that the most isotropic character, according to the data presented in Supplementary Figure S2 (Histograms of fiber orientation in samples), is distinguished for PEO20 NaCl, CS90 + PEO10 NaCl, CS90 + PEO20 NaCl, and CS50 + PEO15 NaCl. 

Table 4 provides information about the mean orientation of fibers in the samples. It can be seen that totally from 2% to 14%, nanofibers are oriented close to the mean orientation: in the sample PEO10 NaCl 11% of fibers were oriented close to 36°, while in the sample CS50 + PEO20 NaCl only 3% of fibers were oriented at 32°.

Also, taking into account the values of the orientation, presented in Table 4, it is clearly seen that fibers in the samples CS90 + PEO15 NaCl, CS50 + PEO10 NaCl, CS50 + PEO15 NaCl, and CS50 + PEO20 NaCl are predominantly aligned horizontally, as the mean orientation value is closer to 0°, while fibers in samples PEO15 NaCl, PEO20 NaCl, and CS90 + PEO20 NaCl are arranged with more vertical alignment (the orientation value is closer to 90).

The diameter of nanofibers holds paramount importance in determining their functionality across various applications such as adsorption, filtration, catalytic degradation, and more. Several factors intricately influence the variation in nanofiber diameters. These factors encompass the concentration of the polymer solution, surface tension, viscosity, conductivity, flow rate, applied voltage, distance between the needle and collector, needle size, and relative humidity. The meticulous adjustment of these parameters is pivotal to fabricating nanofiber membranes possessing the desired diameters and functional properties. Precise control and calibration of these factors are essential to achieve the intended nanofiber diameters, ensuring the attainment of desired functional attributes in the resultant membranes for specific applications. Fine-tuning these parameters is crucial to meet the targeted performance and functionality requirements of nanofiber-based materials [45].

**Figure 6:**
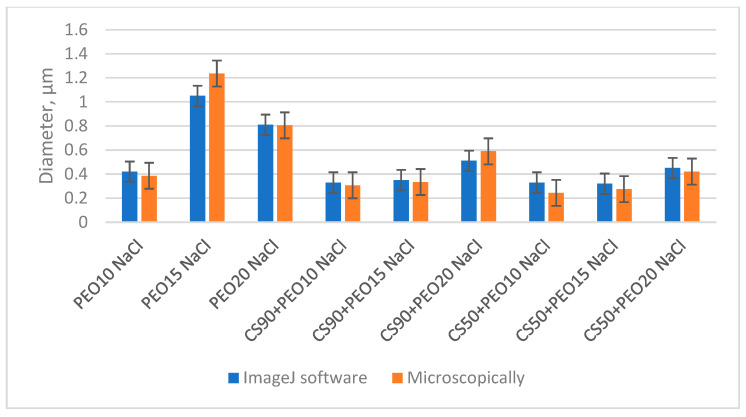
Comparison of the distribution of fiber diameter calculated with ImageJ software and by microscopical analysis.

The distribution of fiber diameters in the samples was assessed through two different methods: microscopic examination and the utilization of ImageJ software, as illustrated in Figure 6. Notably, the values for fiber diameter obtained via software analysis and those measured with a microscope exhibited remarkable similarity. The difference among the results of diameter calculated with software and taken with a microscope varies from 0% to approximately 12%. The detailed diagrams of diameter calculations with ImageJ software are presented in Supplementary Figure S3 (Distribution of fiber diameter, computed with ImageJ). These findings reveal that, with the exception of the PEO15 NaCl sample, very thin fibers falling within the nanofiber range were predominant in all samples. The standard deviation of calculations of nanofibers diameters varied from 0.13 µm till 0.42 µm.

It is worth mentioning that the electrospinning of the PEO15 NaCl solution encountered significant challenges, resulting in the production of notably thick and defective fibers, with an average diameter of roughly 1 µm. Likewise, the diameter of PEO20 NaCl nanofibers was around 800 nm, but microscopical analysis revealed the presence of numerous bead-like structures (as depicted in Figure 5c). These larger diameters observed in the PEO15 NaCl and PEO20 NaCl fibers were primarily attributed to the elevated viscosity of these solutions, as indicated in Table 2.

Furthermore, nanofibers produced from blend solutions containing 50% AA exhibited thinner diameters and narrower diameter distributions compared to those produced from solutions with 90% AA. This variation was mainly influenced by the electrical conductivity and viscosity of the solutions, as detailed in Table 2. Notably, the diameters of nanofibers produced from CS90 + PEO20 NaCl solutions were the largest among the chitosan/PEO solutions, primarily due to the relatively low electrical conductivity (2.9 mS/cm) and high viscosity (2527 cP) of the spinning solution.

## 4. Conclusions

The experiments demonstrated that increase of salt-added PEO concentrations to the CS decreased the electrical conductivity and decreased the viscosity of manufactured solutions. The introduction of salt into chitosan blends had notable effects on the properties of the resulting solutions and fiber mats. The conductivity of the produced solutions and fiber mats was observed to increase as a result of salt addition. The sodium chloride present in the solutions enhanced electrical properties and increased conductivity values more than 50 times for PEO solutions and six times for CS/PEO blend solutions, compared to the conductivity values of solutions without salt. The spinnability of the solutions did not improve, indicating that despite attempting changes or adjustments in the solution composition or processing parameters, the overall capability of the solutions to be successfully processed or spun into nanofibers did not enhance. This lack of improvement suggests that the modifications made did not positively impact the solutions’ ability to form fibers effectively through the electrospinning process. Further optimization or alternative adjustments might be necessary to enhance the spinnability of the solutions for better nanofiber production. During the spinning process, a considerable quantity of wet fibers reached the collector without experiencing sufficient solvent evaporation. This inadequate drying period resulted in the creation of non-round fibers with inconsistent or varying diameters. Insufficient solvent evaporation prevented the fibers from solidifying or stabilizing properly, leading to irregular shapes and diameters as they were collected. This issue suggests the need to optimize the processing parameters or environmental conditions to ensure proper solvent evaporation and the formation of more uniform and rounded fibers. Adjusting parameters like airflow, temperature, or processing speed could potentially mitigate this problem and aid in producing more consistent fibers. Additionally, some undissolved salt crystals were detected within the produced fiber mats. Some ribbon-like fibers were more easily formed as a result of these conditions. Notably, nanofibers produced from a solution containing 50% AA were almost twice thinner and exhibited a narrower diameter distribution compared to those produced from a solution with 90% AA. 

The Fourier-transform infrared (FTIR) analysis indicated that the incorporation of salt did not notably affect the functional groups present in the polymers utilized in the blend. Additionally, calorimetry tests confirmed that all the nanofibers displayed semicrystalline properties. Interestingly, the crystallinity of the nanofibers, along with their melting point, exhibited a decrease when salts were included in the spinning solution. This finding suggests that the introduction of salts had an influence on the crystalline structure and thermal properties of the resulting nanofibers, contributing to a reduction in their crystallinity and melting point.

Microscopic analysis of fiber diameters was compared to data obtained through software analysis, and it was determined that both methods yielded repetitive results, making them interchangeable for fiber analysis. The difference among the results of diameter calculated with software and taken by microscope varied from 0% to approximately 12%. The standard deviation of calculations of nanofiber diameters varied from 0.13 µm to 0.42 µm.

## Figures and Tables

**Table 1:** Coding of blend solutions for electrospinning.

Code of Solution/Produced Fiber Mat	CS Concentration in 90% AA	CS Concentration in 50% AA	PEO Concentration in Water
PEO10 NaCl	-	-	10 wt% + NaCl (0.24 mol/L)
PEO15 NaCl	-	-	15 wt% + NaCl (0.24 mol/L)
PEO20 NaCl	-	-	20 wt% + NaCl (0.24 mol/L)
CS90 + PEO10 NaCl	3 wt%	-	10 wt% + NaCl (0.24 mol/L)
CS90 + PEO15 NaCl	3 wt%	-	15 wt% + NaCl (0.24 mol/L)
CS90 + PEO20 NaCl	3 wt%	-	20 wt% + NaCl (0.24 mol/L)
CS50 + PEO10 NaCl	-	3 wt%	10 wt% + NaCl (0.24 mol/L)
CS50 + PEO15 NaCl	-	3 wt%	15 wt% + NaCl (0.24 mol/L)
CS50 + PEO20 NaCl	-	3 wt%	20 wt% + NaCl (0.24 mol/L)
PEO20	-	-	20 wt%
CS90 + PEO20	3 wt%	-	20 wt%

**Table 2:** Properties of solutions, used for production of fiber mats.

Code of Solution	Conductivity, mS/cm	Viscosity, cP
PEO10 NaCl	16.9	222
PEO15 NaCl	16.5	1745
PEO20 NaCl	7.6	2085
CS90 + PEO10 NaCl	4.9	641
CS90 + PEO15 NaCl	4.5	1485
CS90 + PEO20 NaCl	2.9	2527
CS50 + PEO10 NaCl	7.3	523
CS50 + PEO15 NaCl	5.7	1140
CS50 + PEO20 NaCl	4.9	2210
PEO20	0.14	2300
CS90 + PEO20	0.83	4122

**Table 3:** Thermal properties of nanofibers tested.

Code of Sample	T_mII_ °C	ΔH_mII_, J/g	T_c_ °C	ΔH_c_, J/g	Xc, %
PEO20 NaCl	66.7	118.8	42.2	112.7	55.6
CS50 + PEO20 NaCl	63.4	85.4	42.3	83.1	40.0
CS90 + PEO20 NaCl	63.2	118.6	45.1	126.3	55.5
PEO15 NaCl	66.3	121.1	41.3	116.7	56.7
CS90 + PEO15 NaCl	62.2	120.4	45.9	120.0	56.3
PEO10 NaCl	64.7	120.9	39.0	115.5	56.6
CS50 + PEO10 NaCl	62.8	85.3	45.5	83.2	40.0
CS90 + PEO 10 NaCl	62.6	97.7	46.7	94.5	45.7

**Table 4:** Data about the orientation of nanofibers in the samples.

Code of Sample	Orientation	Coherency
PEO10 NaCl	35.98°	0.11
PEO15 NaCl	−84.36°	0.05
PEO20 NaCl	44.53°	0.07
CS90 + PEO10 NaCl	18.55°	0.07
CS90 + PEO15 NaCl	−11.92°	0.14
CS90 + PEO20 NaCl	58.34°	0.02
CS50 + PEO10 NaCl	−8.85°	0.10
CS50 + PEO15 NaCl	−17.28°	0.04
CS50 + PEO20 NaCl	32.15°	0.03

## Data Availability

The data presented in this study are available upon request from the corresponding author.

## Supplementary Materials

The following supporting information can be downloaded at: https://www.mdpi.com/article/10.3390/ma17010132/s1, Figure S1. Segmented SEM images for determination of fiber diameter distribution with ImageJ; Figure S2. Histograms of fiber orientation and directionality in samples; Figure S3. Distribution of fiber diameter, computed with ImageJ.
