# Supplementary material for: The Investigation of the Production of Salt-Added Polyethylene Oxide/Chitosan Nanofibers
Source: Materials (Basel). 2023 Dec 27;17(1):132. doi: 10.3390/ma17010132 (PMC10779878; doi:10.3390/ma17010132)
Supplement: Supplementary file 1 [file materials-17-00132-s001.zip › materials-2764401-supplementary.pdf]

SUPPORTING INFORMATION

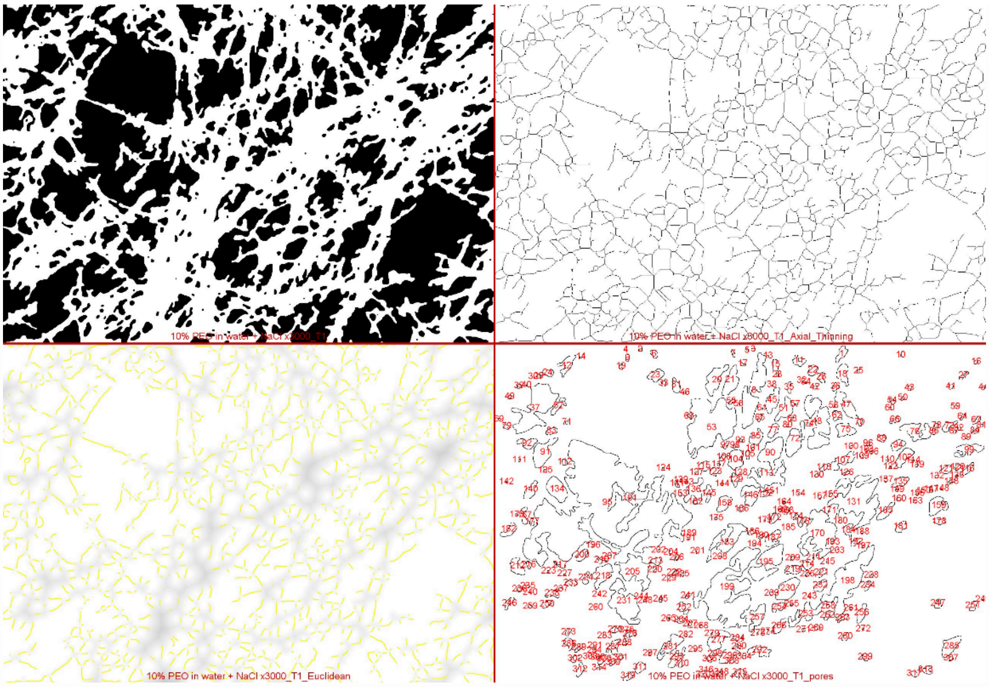

(a)

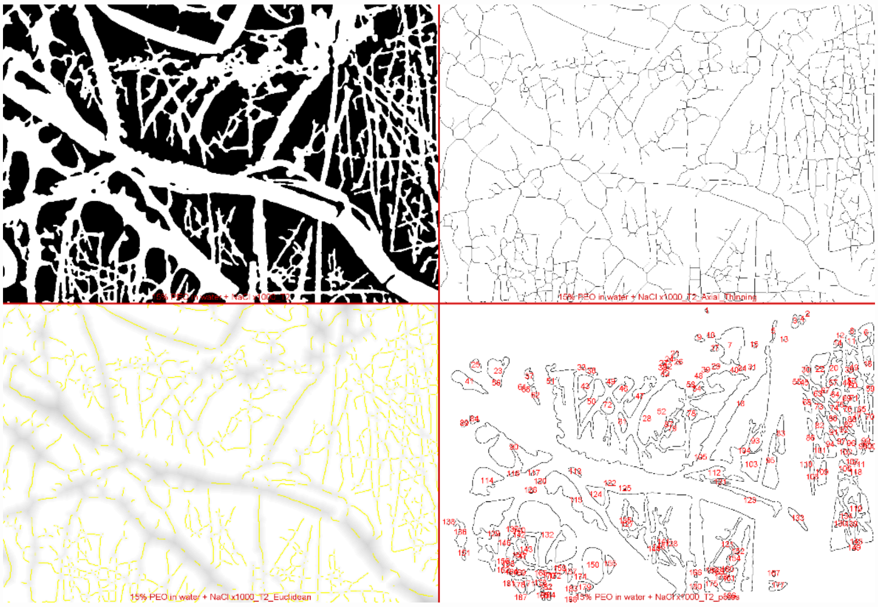

(b)

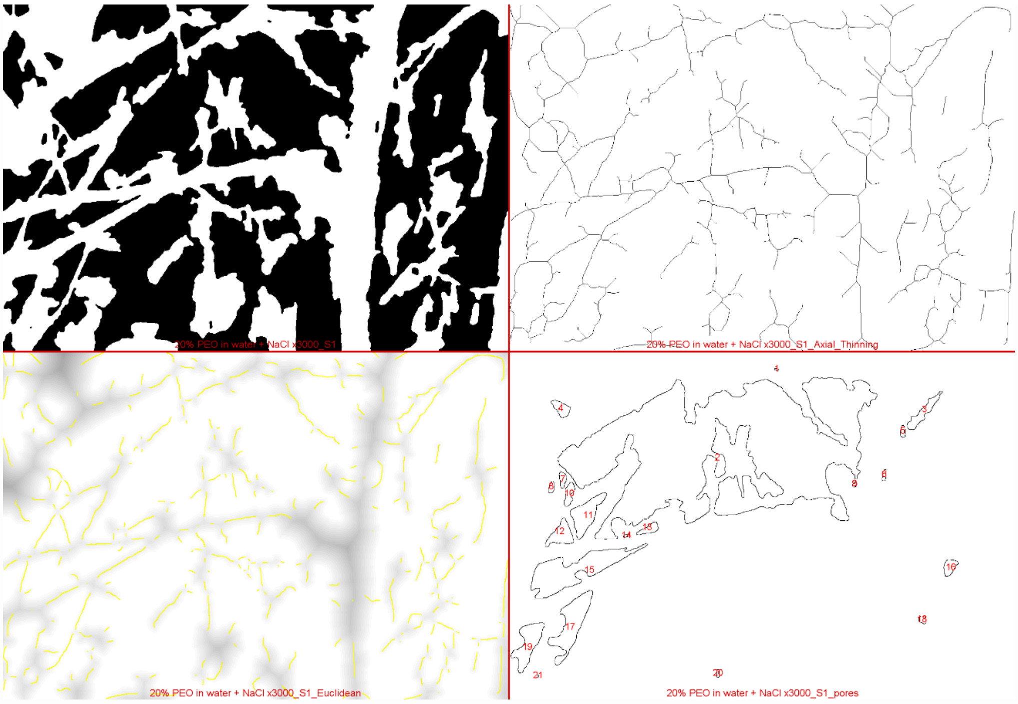
20% PEO in water + NaCl x3000_S1_Axial_Thinning
20% PEO in water + NaCl x3000_S1_Euclidean
20% PEO in water + NaCl x3000_S1_pores

(c)

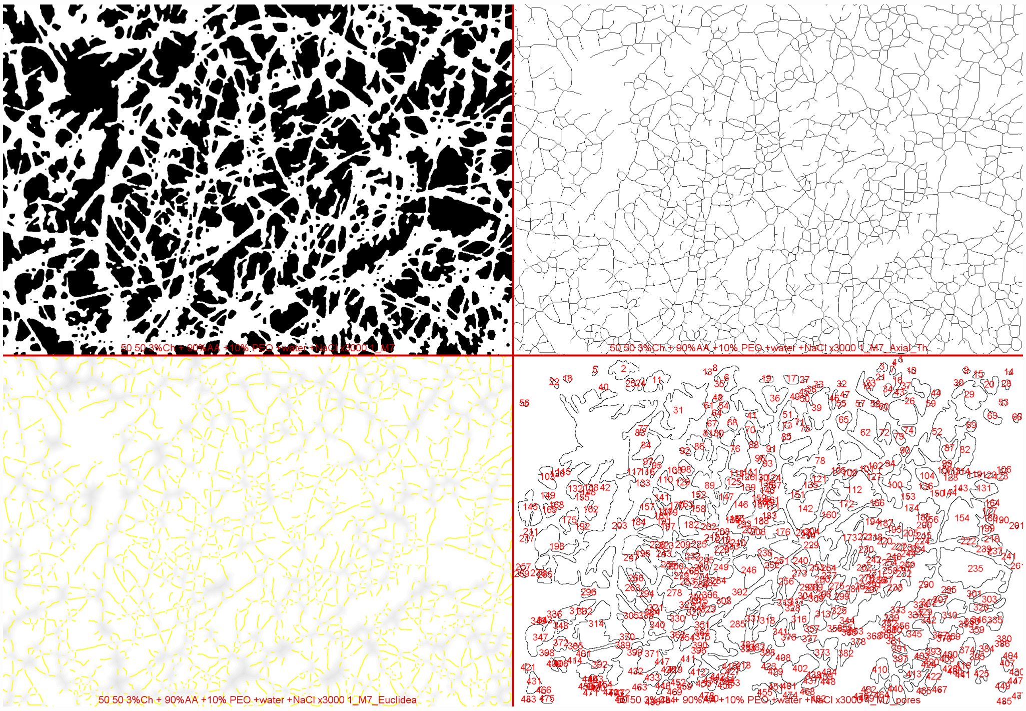
50 50 3%Ch + 90%AA +10% PEO +water +NaCl x3000 1_M7_Euclidea

(d)

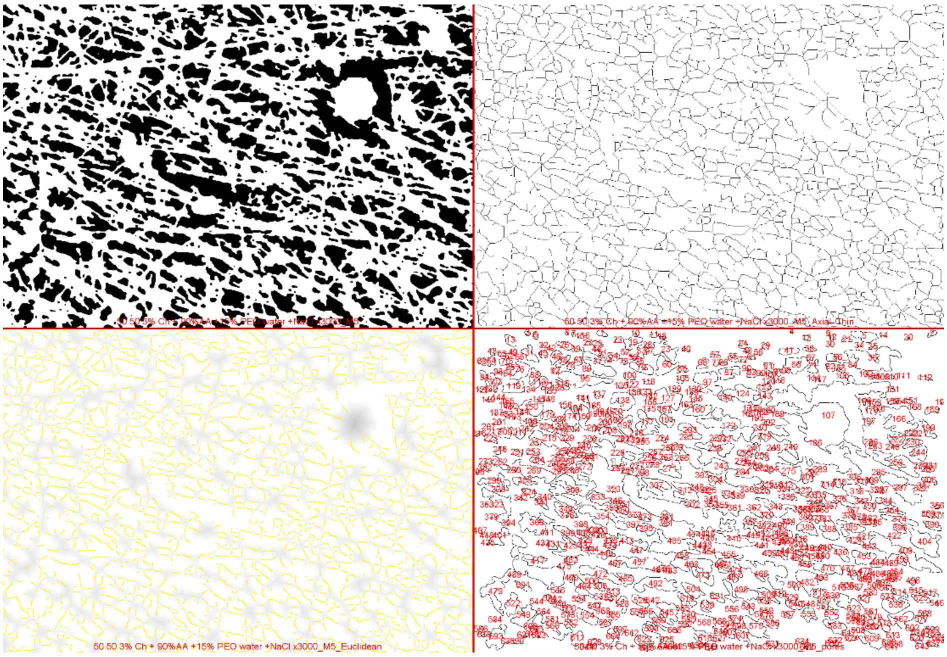
50 50 3% Ch + 90%AA +15% PEO water +NaCl x3000_M5_Euclidean

(e)

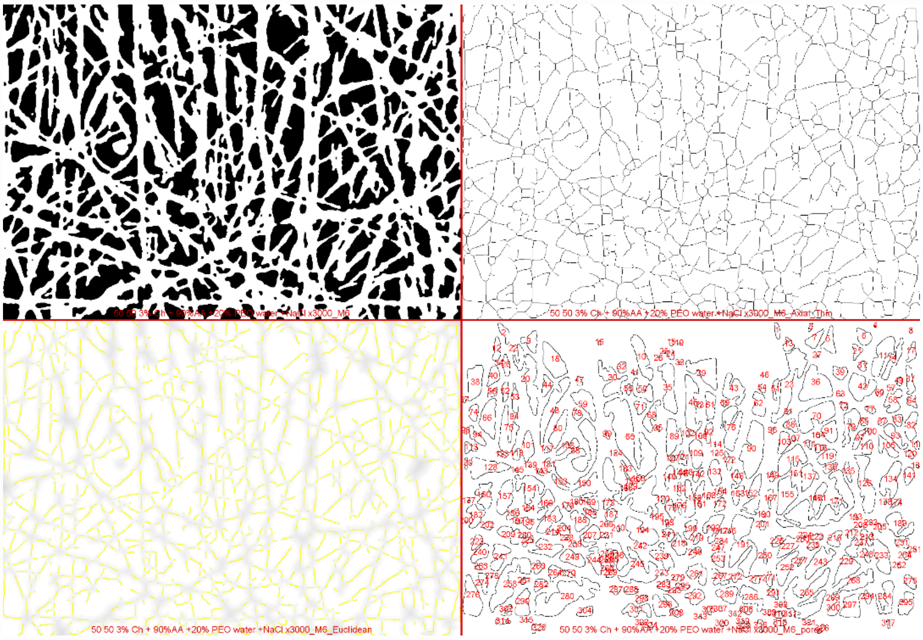
50 50 3% Ch + 90%AA +20% PEO water +NaCl x3000_M6_Axial_Thin
50 50 3% Ch + 90%AA +20% PEO water +NaCl x3000_M6_Euclidean

(f)

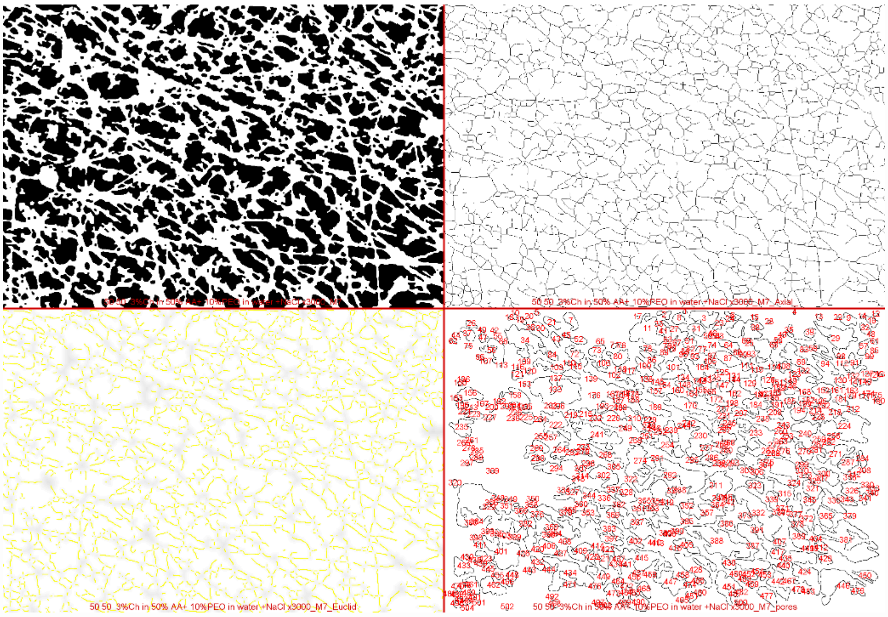
50 50_3%Ch in 50% AA+ 10%PEO in water +NaCl x3000_M7_Axial
50 50_3%Ch in 50% AA+ 10%PEO in water +NaCl x3000_M7_Euclid

(g)

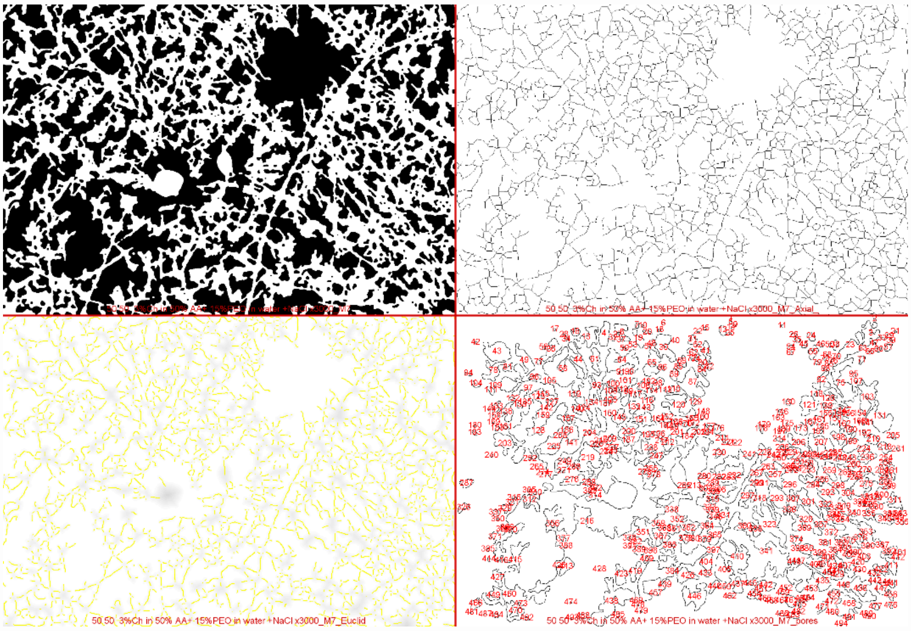
50 50_3%Ch in 50% AA+ 15%PEO in water +NaCl x3000_M7_Axial
50 50_3%Ch in 50% AA+ 15%PEO in water +NaCl x3000_M7_Euclid
50 50_3%Ch in 50% AA+ 15%PEO in water +NaCl x3000_M7_pores

(h)

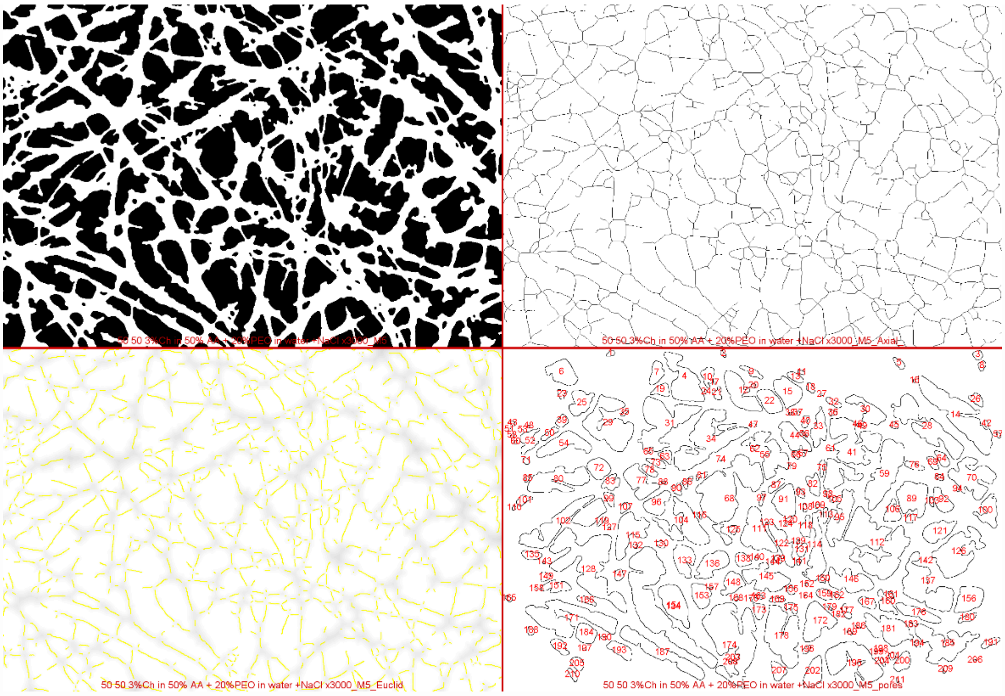

(i)

Figure S1. Segmented SEM images for determination of fiber diameter distribution with ImageJ: a) PEO10 NaCl; b) PEO15 NaCl; c) PEO20 NaCl; d) CS90+PEO10 NaCl; e) CS90+PEO15 NaCl; f) CS90+PEO20 NaCl; g) CS50+PEO10 NaCl; h) CS50+PEO15 NaCl; i) CS50+PEO20 NaCl.

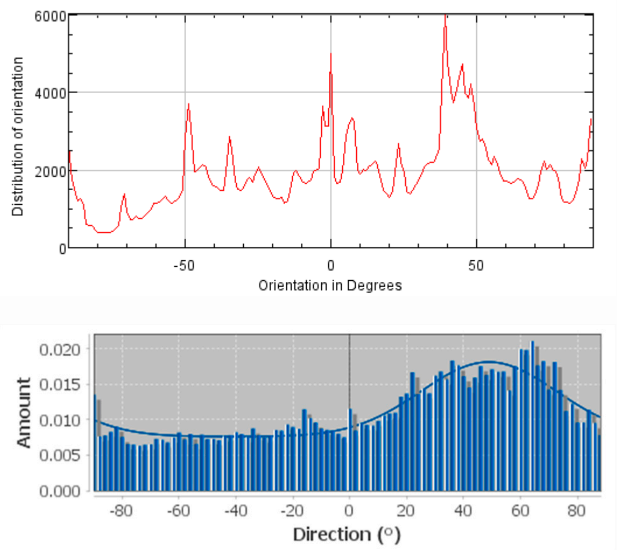

(a)

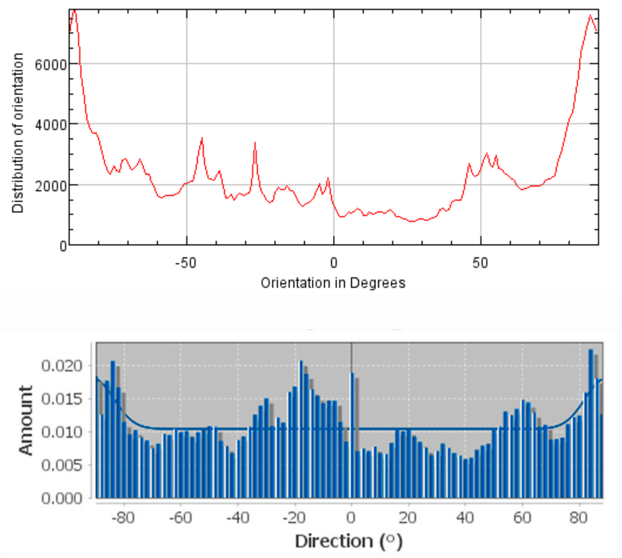
Distribution of orientation
0
2000
4000
6000
-50
0
50
Orientation in Degrees
Amount
0.000
0.005
0.010
0.015
0.020
-80
-60
-40
-20
0
20
40
60
80
Direction (°)

(b)

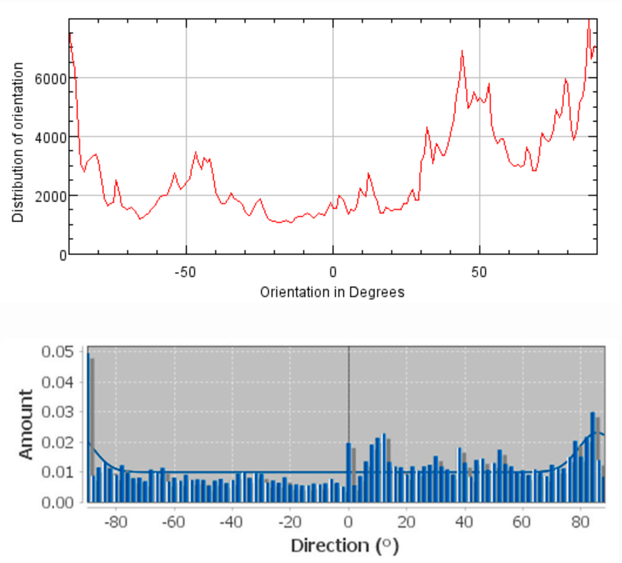
Distribution of orientation
0
2000
4000
6000
-50
0
50
Orientation in Degrees
Amount
0.00
0.01
0.02
0.03
0.04
0.05
-80
-60
-40
-20
0
20
40
60
80
Direction (°)

(c)

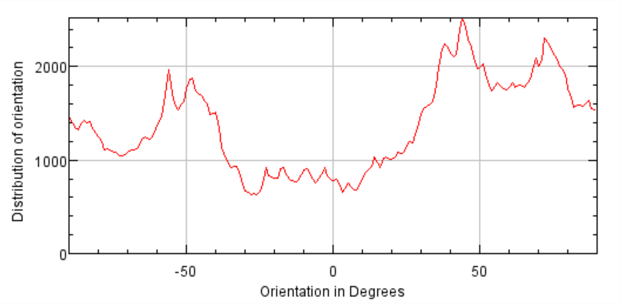
Distribution of orientation
0
1000
2000
-50
0
50
Orientation in Degrees

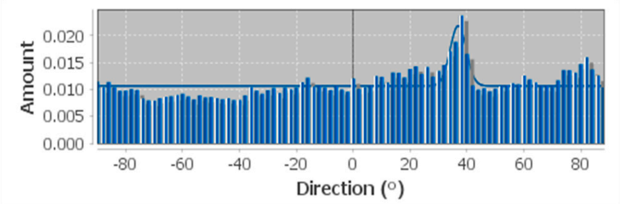

Amount
0.020
0.015
0.010
0.005
0.000
-80
-60
-40
-20
0
20
40
60
80
Direction (°)

(d)

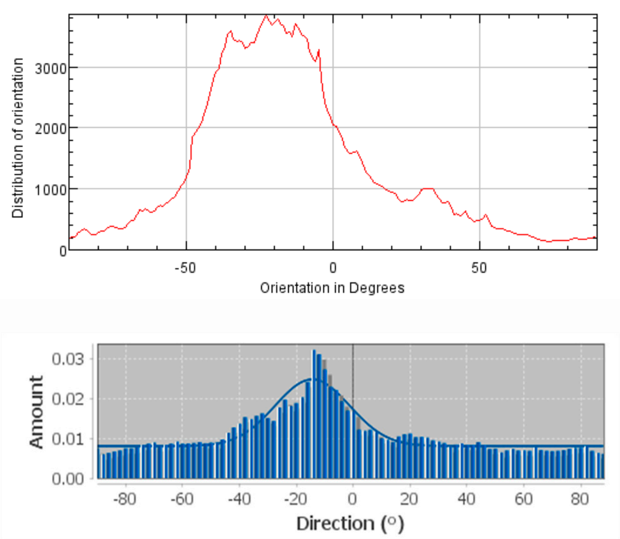

Distribution of orientation
3000
2000
1000
0
-50
0
50
Orientation in Degrees
Amount
0.03
0.02
0.01
0.00
-80
-60
-40
-20
0
20
40
60
80
Direction (°)

(e)

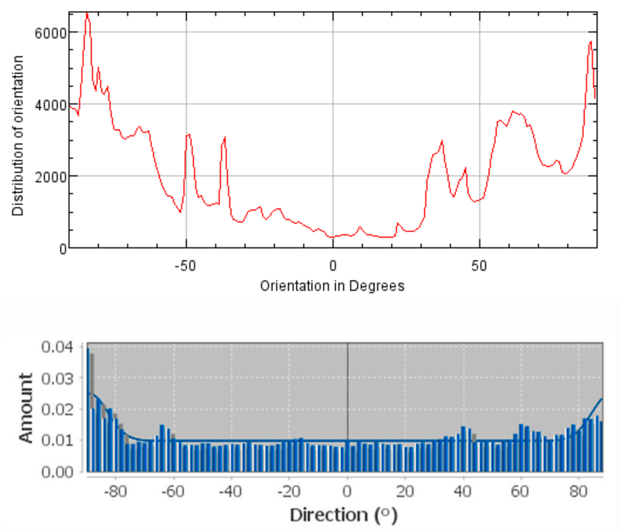

Distribution of orientation
6000
4000
2000
0
-50
0
50
Orientation in Degrees
Amount
0.04
0.03
0.02
0.01
0.00
-80
-60
-40
-20
0
20
40
60
80
Direction (°)

(f)

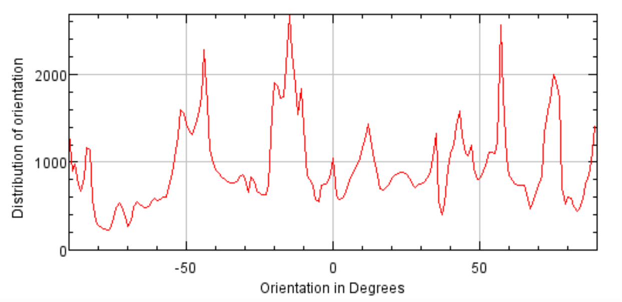
Distribution of orientation
2000
1000
0
-50
0
50
Orientation in Degrees

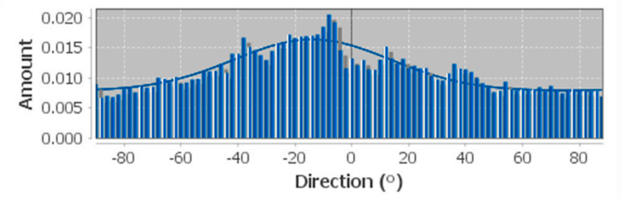
Amount
0.020
0.015
0.010
0.005
0.000
-80
-60
-40
-20
0
20
40
60
80
Direction (°)

(g)

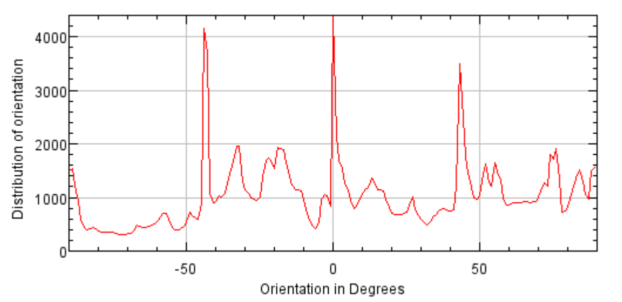
Distribution of orientation
4000
3000
2000
1000
0
-50
0
50
Orientation in Degrees

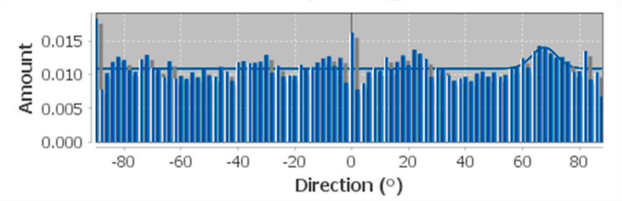
Amount
0.015
0.010
0.005
0.000
-80
-60
-40
-20
0
20
40
60
80
Direction (°)

(h)

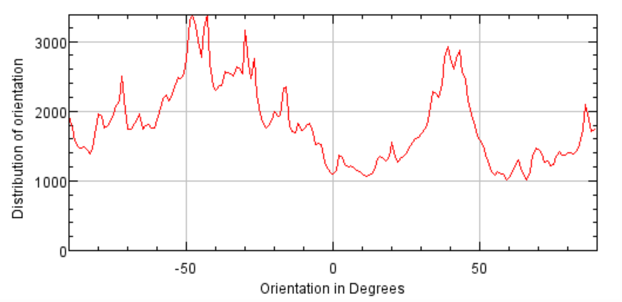
Distribution of orientation
3000
2000
1000
0
-50
0
50
Orientation in Degrees

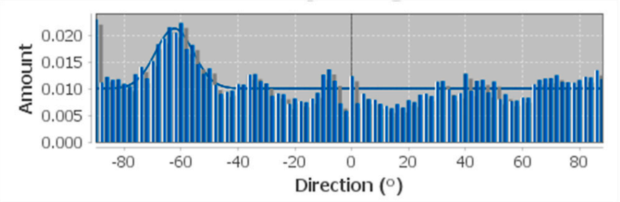

(i)

Figure S2. Histograms of fiber orientation and directionality in samples: a) PEO10 NaCl, b) PEO15 NaCl, c) PEO20 NaCl, d) CS90+PEO10 NaCl e) CS90+PEO15 NaCl, f) CS90+PEO20 NaCl, g) CS50+PEO10 NaCl, h) CS50+PEO15 NaCl, i) CS90+PEO20 NaCl

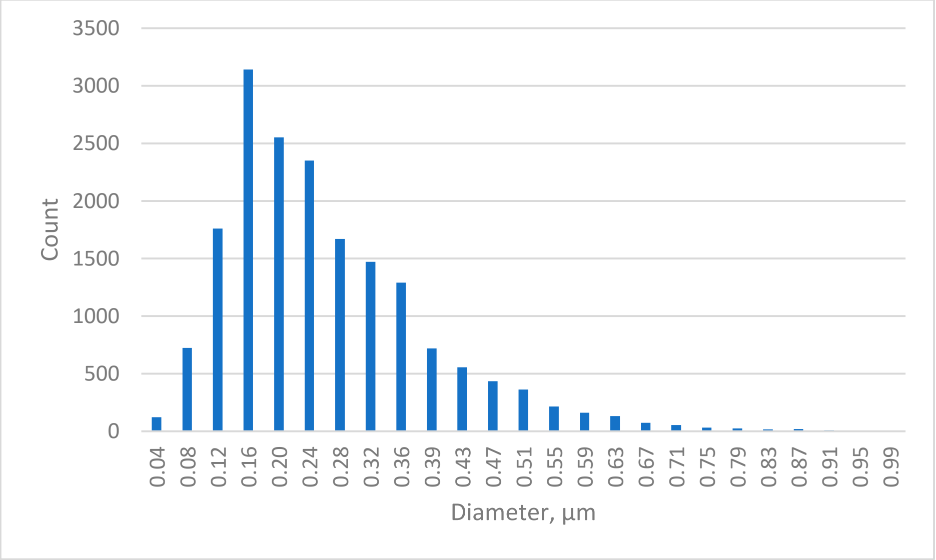

(a)

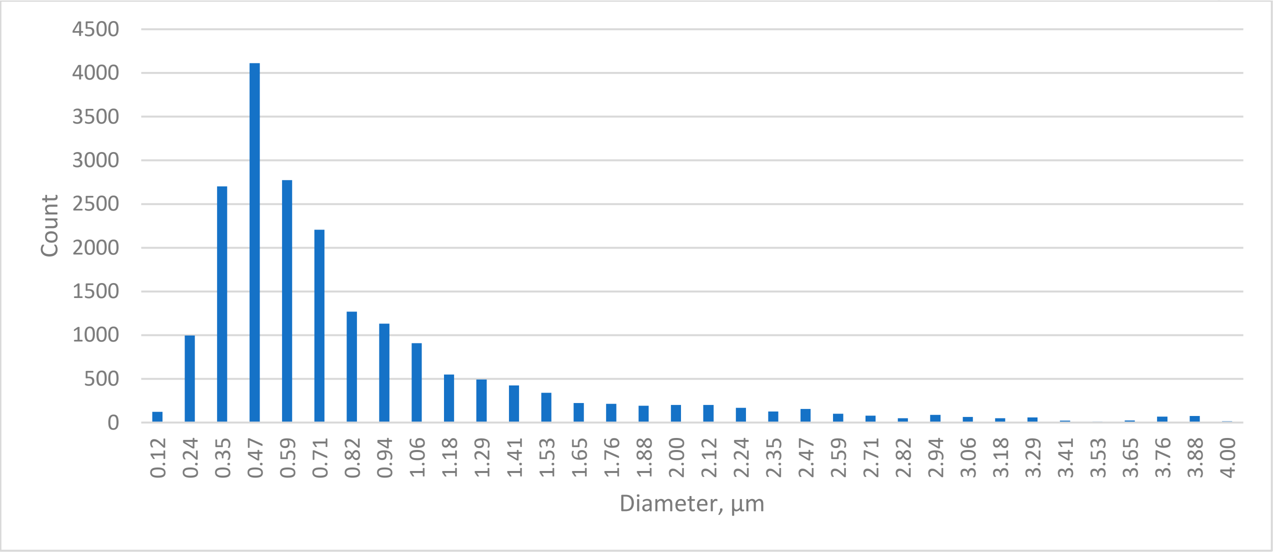

(b)

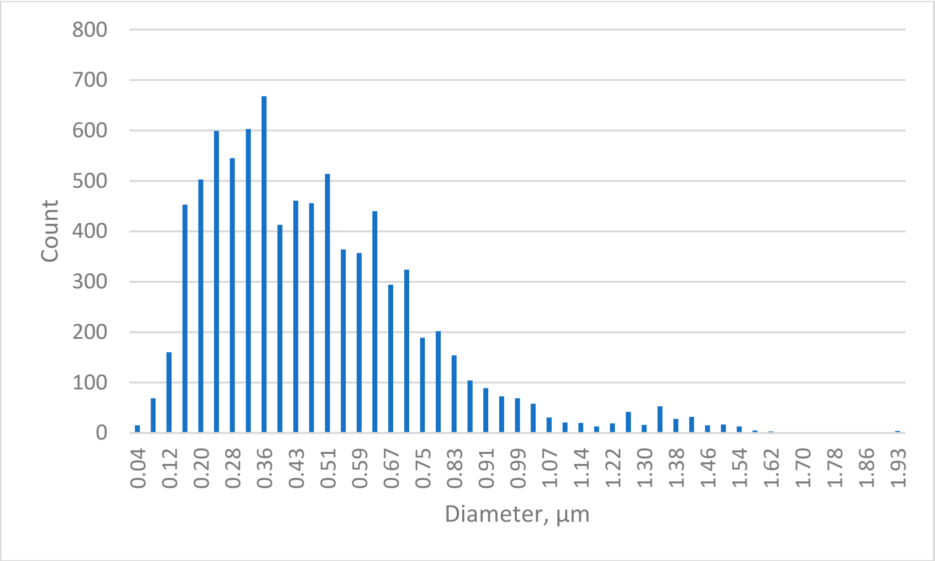

(c)

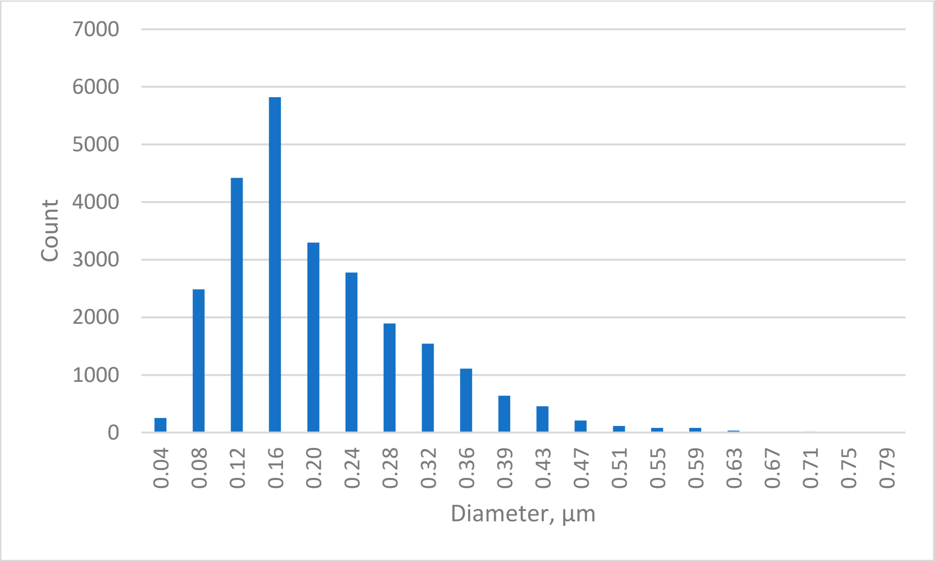

7000
6000
5000
4000
3000
2000
1000
0
Count
0.04 0.08 0.12 0.16 0.20 0.24 0.28 0.32 0.36 0.39 0.43 0.47 0.51 0.55 0.59 0.63 0.67 0.71 0.75 0.79
Diameter, µm

(d)

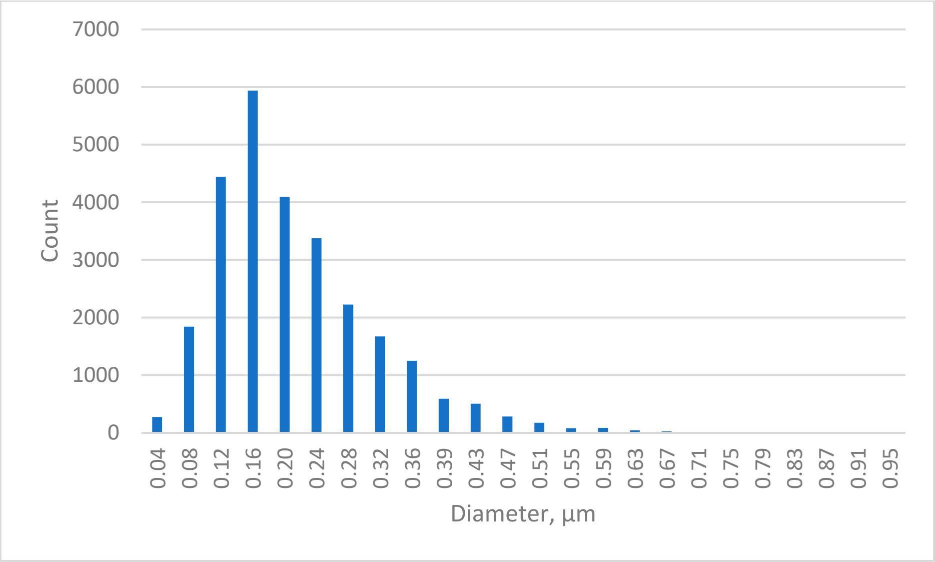

7000
6000
5000
4000
3000
2000
1000
0
Count
0.04 0.08 0.12 0.16 0.20 0.24 0.28 0.32 0.36 0.39 0.43 0.47 0.51 0.55 0.59 0.63 0.67 0.71 0.75 0.79 0.83 0.87 0.91 0.95
Diameter, µm

(e)

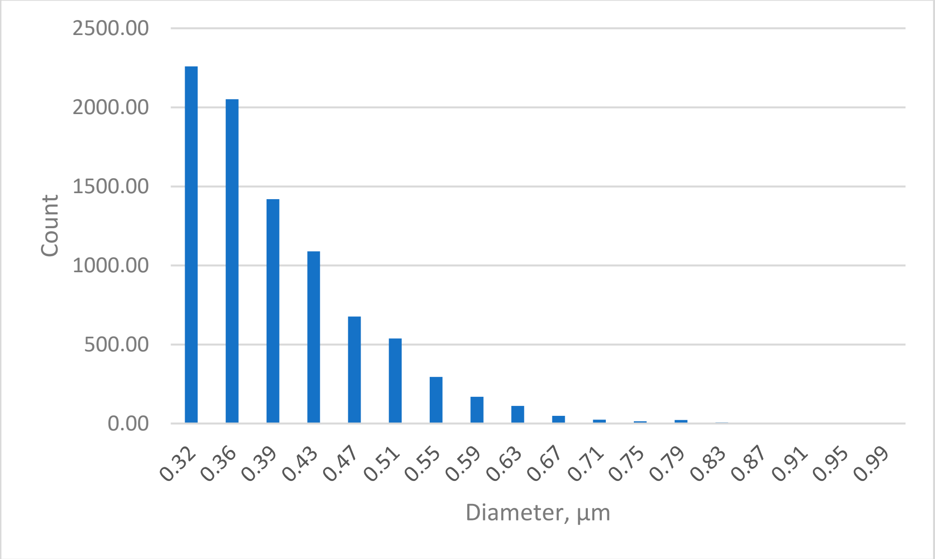
2500.00
2000.00
1500.00
1000.00
500.00
0.00
Count
0.32 0.36 0.39 0.43 0.47 0.51 0.55 0.59 0.63 0.67 0.71 0.75 0.79 0.83 0.87 0.91 0.95 0.99
Diameter, µm

(f)

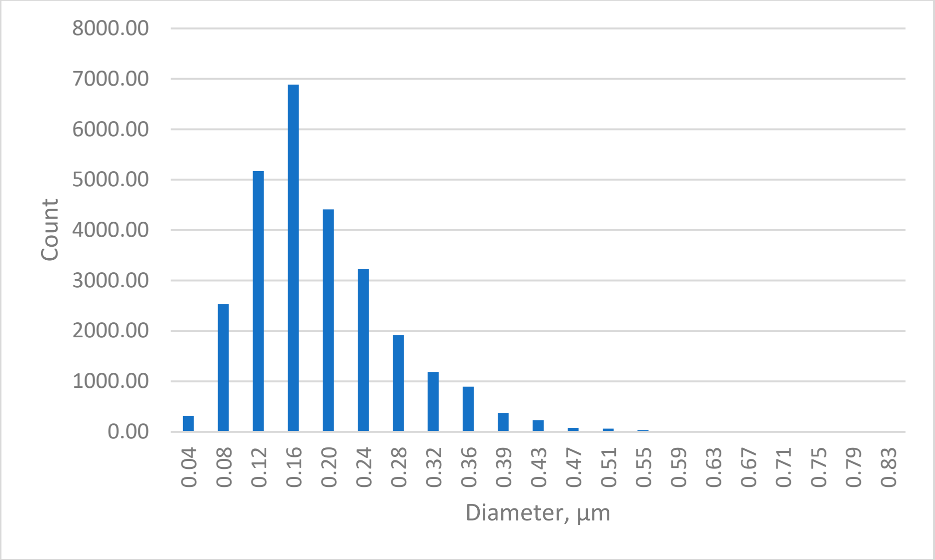
8000.00
7000.00
6000.00
5000.00
4000.00
3000.00
2000.00
1000.00
0.00
Count
0.04 0.08 0.12 0.16 0.20 0.24 0.28 0.32 0.36 0.39 0.43 0.47 0.51 0.55 0.59 0.63 0.67 0.71 0.75 0.79 0.83
Diameter, µm

(g)

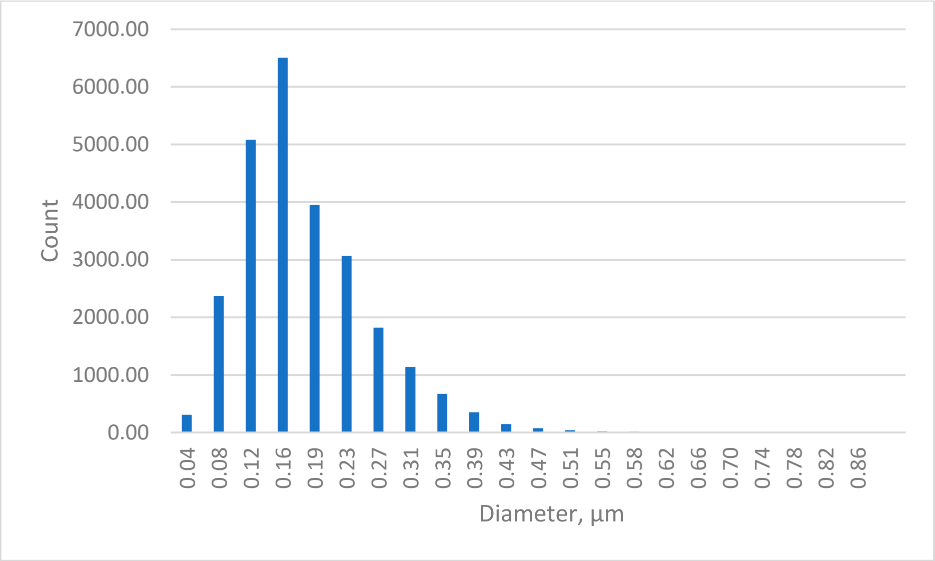

(h)

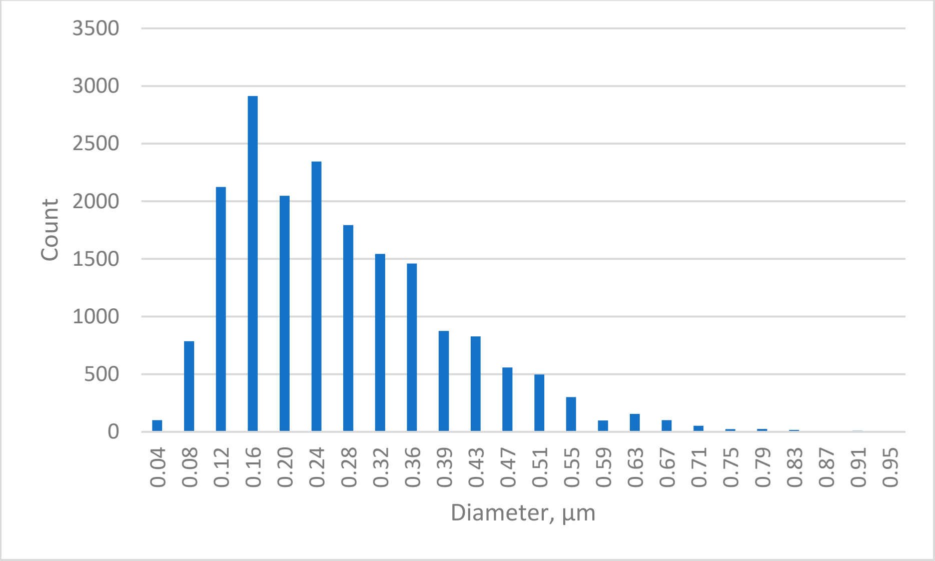

(i)

Figure S3. Distribution of fiber diameter, computed with ImageJ: a) PEO10 NaCl (mean diameter = 0.42 µm, STD = 0.20 µm), b) PEO15 NaCl (mean diameter = 1.05 µm, STD = 0.42 µm), c) PEO20 NaCl (mean diameter = 0.81 µm, STD = 0.47 µm), d) CS90+PEO10 NaCl (mean diameter = 0.33 µm, STD = 0.15 µm), e) CS90+PEO15 NaCl (mean diameter = 0.35 µm, STD = 0.15 µm), f) CS90+PEO20 NaCl (mean diameter = 0.51 µm, STD = 0.23 µm), g) CS50+PEO10 NaCl (mean diameter = 0.33 µm, STD = 0.13 µm), h) CS50+PEO15 NaCl (mean diameter = 0.32 µm, STD = 0.13 µm), i) CS50+PEO20 NaCl (mean diameter = 0.45 µm, STD = 0.24 µm)
